# LncRNA SENCR suppresses abdominal aortic aneurysm formation by inhibiting smooth muscle cells apoptosis and extracellular matrix degradation

**DOI:** 10.17305/bjbms.2020.4994

**Published:** 2021-06

**Authors:** Zhou Cai, Jianhua Huang, Junxiao Yang, Baihong Pan, Wei Wang, Yangyang Ou, Xianwei Wang, Pu Yang

**Affiliations:** 1 Department of General Surgery, Xiangya Hospital Central South University, Changsha, China; 2 Department of Orthopedics, Xiangya Hospital Central South University, Changsha, China

**Keywords:** Abdominal aortic aneurysm, lncRNA SENCR, vascular smooth muscle cells, cell apoptosis, extracellular matrix degradation

## Abstract

Abdominal aortic aneurysm (AAA) is a progressive chronic dilatation of the abdominal aorta without effective medical treatment. This study aims to clarify the potential of long non-coding RNA SENCR as a treatment target in AAA. Angiotensin II (Ang-II) was used to establish AAA model *in vitro* and *in vivo*. Reverse transcription quantitative PCR and western blot were performed to measure the expression of SENCR and proteins, respectively. Annexin V-FITC/PI double staining was carried out to detect the apoptotic rate in vascular smooth muscle cells (VSMCs), and cell apoptosis in aortic tissues was determined by TUNEL staining. Besides, hematoxylin and eosin and Elastica van Gieson staining were performed for histological analysis of aortic tissues. SENCR was downregulated in AAA tissues and Ang-II-stimulated VSMCs. Overexpression of SENCR could inhibit Ang-II-induced VSMC apoptosis, while inhibition of SENCR facilitated Ang-II-induced VSMC apoptosis. Moreover, the expression of matrix metalloproteinase (MMP)-2 and MMP-9 in Ang-II-induced VSMCs was reduced following SENCR overexpression, while tissue inhibitor of metalloproteinases 1 (TIMP-1) expression was increased. *In vivo*, overexpression of SENCR improved the pathological change in aortic tissues and the damage in arterial wall elastic fibers induced by Ang-II, as well as suppressed Ang-II-induced cell apoptosis and extracellular matrix degradation. Overall, SENCR was decreased in AAA. Overexpression of SENCR inhibited AAA formation via inhibition of VSMC apoptosis and extracellular matrix degradation. We provided a reliable evidence for SENCR acting as a potential target for AAA treatment.

## INTRODUCTION

Abdominal aortic aneurysm (AAA) is defined as a maximal infrarenal abdominal aortic diameter more than 30 mm or at least 1.5 times the normal diameter, and the pathologic changes of abdominal aorta characterized by permanent and irreversible dilation [1,2]. AAA is typically asymptomatic until rupture occurs [3]. Rupture leads to death in 65% patients with AAA. Family history, male gender, smoking habit, and especially age above 65 years are the major risk factors for AAA formation and development. The prevalence of AAA is increasing with the increased age of patients [1,4]. No effective biomarker has been used for the diagnosis of AAA, and open surgical repair is still the primary therapeutic method of the disease [5]. Numerous animal models, such as angiotensin II (Ang-II)-induced AAA mouse model, were developed to explore more treatment methods for the disease [6-8]. Several lines of evidence have revealed that the number of smooth muscle cells (SMCs) in AAA tissues is significantly reduced, and the pathogenesis of AAA is closely associated with inflammation in the aortic wall and extracellular matrix degeneration [9,10]. Hence, it is necessary to investigate the pathological mechanism of AAA and find new molecular targets for AAA treatment.

Long non-coding RNAs (lncRNAs) are a class of RNA transcripts with more than 200 nucleotides [11]. In the past 10 years, more and more lncRNAs were recognized as new regulators of gene expression, and they play an important role in the occurrence and development of multiple disorders [12,13]. In recent years, lncRNAs were also proved to be crucial in the progression of AAA. For instance, Yang et al. found that the number of abnormally expressed lncRNAs is up to 3688, and some lncRNAs, like lnc-ARG, are involved in the pathogenesis of AAA [14].

However, in AAA progression, only a few lncRNAs were explored. Smooth muscle and endothelial cell enriched migration/differentiation-associated lncRNA (SENCR) is a recently discovered lncRNA, enriched in vascular SMCs (VSMCs) and endothelial cells [15]. It was demonstrated that SENCR regulates the proliferation, migration and phenotype of SMCs, and knockdown of SENCR could effectively promote the proliferation of SMCs and the development of atherosclerosis [16,17]. Interestingly, AAA has a number of similar pathological characteristics to atherosclerosis. AAA is becoming widely recognized as a response to atherosclerosis [18,19]. Yu et al. directly suggested that dysregulated SENCR is associated with the progression of atherosclerotic vascular disorder [20]. However, the role of SENCR in AAA progression remains unclear. In this present study, we found that SENCR was decreased in both AAA tissues and Ang-II-induced VSMCs. Overexpression of SENCR could notably inhibit the formation of AAA through the inhibition of VSMC apoptosis and degradation of extracellular matrix.

## MATERIALS AND METHODS

### AAA mouse model

All animal experiments were approved by the Institutional Animal Care and Use Committee of Xiangya Hospital Central South University and were carried out according to the guidelines for the Care and Use of Laboratory Animals of the National Institutes of Health. Apolipoprotein E-deficient (ApoE^-/-^) mice, on a C57BL/6J genetic background (male, 12 weeks old), were obtained from Shanghai SLAC Laboratory Animal Co., Ltd. (Shanghai, China), and then were raised in a safe environment with a 12-hour light/dark cycle. To establish AAA mouse model, ApoE^-/-^ mice were infused with Ang-II (Sigma-Aldrich, St. Louis, MO, USA) at a rate of 1 μg/kg/min during the course of 28 days using a minipump (Alzet Osmotic Pump, Model 2004; Durect Corp, USA) [21]. In the normal group, ApoE^-/-^ mice were infused with equal volume of 0.9% NaCl. Then, the expression of SENCR in aortic tissues was measured using reverse transcription quantitative polymerase chain reaction (RT-qPCR) at 0, 7, 14, 21, and 28 days after Ang-II or 0.9% NaCl induction.

### RT-qPCR analysis

Total RNA was isolated from aortic tissues or VSMCs utilizing TRIzol reagent (Invitrogen, Carlsbad, CA) and subsequent reverse transcribed into cDNA using a Reverse Transcription System Kit (TaKaRa Biotechnology, Dalian, China) in accordance with the specific manufacturer’s instructions. Next, quantitative PCR was carried out with a SYBR Premix Ex Taq™ kit (Invitrogen) according to the protocol provided on the system of Light Cycler 480 II (Roche Diagnostics, Basel, Switzerland). Here, the relative expression of SENCR was normalized to β-actin, and calculated using the 2^−DDCt^ method. The primer sequences were as follows: SENCR: 5’-CAGCCAGAAAGGACTCCAACTCC-3’ (F’) and 5’-GGAG GCAGCTGGTGCTGAAAG-3’ (R’); β-actin: 5’-CATCGTCCACCGCAAATGCTTC -3’ (F’) and 5’- AACCGACTGCTGTCACCTTCAC-3’ (R’).

### Cell culture and Ang-II induction

Mouse aortic VSMCs were obtained from Procell Co. (Wuhan, China). Fetal bovine serum (FBS, Gibco BRL, USA),penicillin, and streptomycin were added into Dulbeccos modified Eagles medium ([DMEM], Gibco BRL). The concentration of FBS, penicillin and streptomycin are 10%, 100 U/mL and 100 g/mL, respectively. Then, VSMCs were cultured in complete DMEM medium at 37°C in a humidified air with 5% CO_2_. Different concentrations of Ang-II (0, 1, 5, and 10 mM) were used to stimulate VSMCs for 0, 12, 24, and 48 hours. Next, the expression of SENCR was measured by RT-qPCR.

### Cell transfection

Full length mouse SENCR cDNA was cloned into pcDNA-3.1 vector and stored in -20°C. Small interfering RNA (siRNA) against SENCR (si-SENCR) or siRNA negative control (si-NC, si-Ctrl) were designed and purchased from Guangzhou RiboBio Co., Ltd. (Guangzhou, China). Next, pcDNA-SENCR (2 μg), empty vector (2 μg), si-SENCR (50 nM), and si-NC (50 nM) were transfected into Ang-II-induced VSMCs using Lipofectamine^®^ 2000 (Invitrogen).

### Analysis of VSMC apoptosis

At 48 hours after transfection, apoptosis rate of VSMCs was measured by Annexin V-FITC/propidium iodide (PI) double staining assay. Here, an Annexin V-FITC/PI apoptosis detection kit (BestBio, Shanghai, China) was used to determine the apoptosis rate of VSMCs on the FACSCanto II flow cytometer (Becton Dickinson, USA), according to the manufacturer’s protocol.

### Western blot analysis

Total proteins were isolated from VSMCs or mouse aortic tissues using RIPA lysis buffer (Santa Cruz Biotechnology, Inc., USA). Equal 25 μg protein lysates were separated on 12% sodium dodecyl sulfate-polyacrylamide gel electrophoresis gel, and then were transferred into polyvinylidene fluoride membranes (Millipore Corp., USA). Next, all membranes were incubated with 5% non-fat milk for 1 hour at room temperature, and then incubated with primary antibodies against matrix metalloproteinase (MMP)-2 (1: 2000, Abcam, Cambridge, USA), MMP-9 (1: 2000, Abcam) and tissue inhibitor of metalloproteinases 1 [TIMP-1] (1:1000, Abcam) overnight at 4°C. Subsequently, all membranes were incubated with secondary antibody (1:3000, Abcam) for 1 hour at room temperature. Finally, an enhanced chemiluminescence kit was utilized to display the protein bands, and all bands were then analyzed using Image J software. The expression of MMP-2, MMP-9 and TIMP-1 were normalized to glyceraldehyde 3-phosphate dehydrogenase (GAPDH).

### Injection of lentivirus (LV)

Empty vector or pcDNA-SENCR was packaged into LV, which was purchased from Vigene Biosciences (Jinan, China). ApoE^-/-^ mice were randomly divided into four groups: saline group (normal group), Ang-II group (AAA group), Ang-II + LV-NC group (LV-NC group), and Ang-II + LV-SENCR group (LV-SENCR group). In Ang-II + LV-NC and Ang-II + LV-SENCR groups, mice were injected with LV carrying pcDNA-SENCR or empty vector (1 × 10^11^ plaque-forming unit [pfu]/mouse) by tail-intravenous injection. At 28 days, aortic tissues were isolated from each mouse, and then the incidence and maximum diameter of AAA were analyzed.

### Histological analysis

Mouse aortic tissues were isolated and fixed with 4% paraformaldehyde at 4°C for 24 hours. Then, aortic tissues were dehydrated and embedded in paraffin. Paraffin sections (6 μm thick) were used to carry out hematoxylin-eosin (HE) and Elastica van Gieson (EVG) staining according to the manufacturer’s instructions. HE staining kit and EVG staining kit were obtained from BOSTER Biological Technology, Co., Ltd. (Wuhan, China) and Abcam, respectively.

### Analysis of apoptotic cells in aortic tissues

TUNEL staining was performed to detect the rate of apoptotic cells in mouse aortic tissues. Tissues were paraffin-embedded and then cut into serial sections. After dewaxing and hydration, the sections were incubated with 50 μl TUNEL working solution (Roche Diagnostics, Germany) at 37°C for 1 hour in a humidifying box. Then, the sections were maintained with 50 μl converter-POD solution at 37°C for 30 minutes followed by incubation with 50 μl DAB solution at room temperature for 10 minutes. Finally, hematoxylin was used to mark the nucleus of cells, and the apoptotic cells were analyzed under a fluorescence microscope (Nikon Corporation, Japan).

### Statistical analysis

IBM SPSS Statistics for Windows, Version 20.0. (IBM Corp., Armonk, NY, USA) was used for data analysis. All data were presented as mean ± standard deviation (SD). The statistical analyses between two independent groups were analyzed by Student’s *t*-test, and the statistical analyses among multiple groups were performed by one-way analysis of variance (ANOVA) followed by Bonferroni’s test. The morbidity of aneurysm was analyzed using the Fisher’s exact test. The statistical significance was recognized as *p* value lower than 0.05. All experiments were repeated 3 times at least.

## RESULTS

### SENCR is downregulated in AAA tissues

**FIGURE 1:**
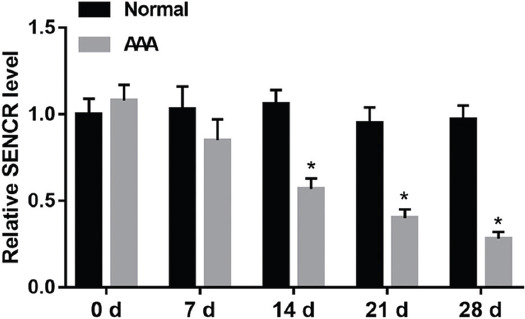
Expression of SENCR in abdominal aortic aneurysm (AAA) tissues. Angiotensin II (Ang-II) was used to establish AAA mouse model. At 0, 7, 14, 21, and 28 days after induction, reverse transcription quantitative polymerase chain reaction (RT-qPCR) was performed to detect the expression of SENCR in AAA tissues and normal aortic tissues. *p < 0.05 compared with normal group. SENCR: Smooth muscle and endothelial cell enriched migration/differentiation-associated lncRNA.

To explore the expression level of SENCR in AAA tissues, Ang-II was injected into ApoE^-/-^ mice to establish AAA animal model, and equal volume of normal saline was injected into ApoE^-/-^ mice to obtain control group. Then, aortic tissues were stripped from mice at 0, 7, 14, 21, and 28 days after Ang-II or normal saline stimulation. The RT-qPCR results revealed that SENCR was notably downregulated in the aortic tissues from AAA mice at 14 days after induction (Figure 1).

### Upregulation of SENCR suppressed Ang-II-induced VSMC apoptosis

**FIGURE 2:**
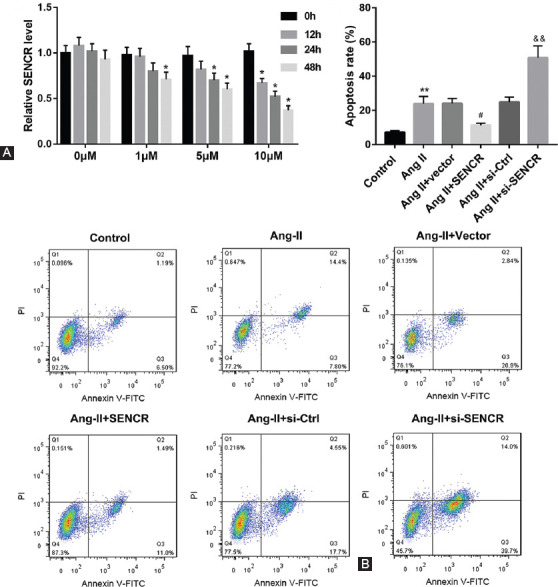
Effect of SENCR on angiotensin II (Ang-II)-induced apoptosis of vascular smooth muscle cells (VSMCs). (A) 0 μM, 1 μM, 5 μM, and 10 μM Ang-II was used to stimulate VSMCs for 0, 12, 24, and 48 hours. Reverse transcription quantitative polymerase chain reaction (RT-qPCR) was carried out to measure the expression of SENCR in VSMCs. *p < 0.05 compared with 0 hour group. (B) Annexin V-FITC/PI double staining assay was performed to examine the apoptotic rate of cells. **p < 0.01 compared with control group, ^#^*p* < 0.05 compared with Ang-II group, and ^&&^*p* < 0.01 compared with Ang-II group. SENCR: Smooth muscle and endothelial cell enriched migration/differentiation-associated lncRNA.

We further explored the effect of SENCR on VSMC apoptosis. Our results demonstrated that Ang-II reduced the expression of SENCR in VSMCs in time- and dose-dependent manners (Figure 2A). Then, we used 10 μM Ang-II to stimulate VSMCs transfected with pcDNA-SENCR, empty vector, si-NC, or si-SENCR. At 48 hours later, we found that overexpression of SENCR significantly inhibited Ang-II-induced VSMC apoptosis, while inhibition of SENCR remarkably facilitated Ang-II-induced apoptosis of VSMCs (Figure 2B).

### Overexpression of SENCR inhibited Ang-II-induced extracellular matrix degradation of VSMCs

**FIGURE 3:**
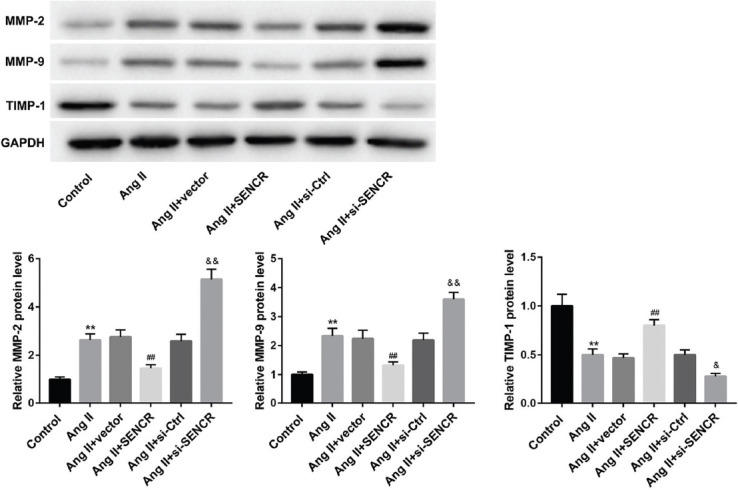
Effect of SENCR on angiotensin II (Ang-II)-induced extracellular matrix degradation in vascular smooth muscle cells (VSMCs). Ang-II (10 μM) was used to stimulate VSMCs, which were then transfected with pcDNA-SENCR, empty vector, si-NC, or si-SENCR. After 48 hours, western blot was performed to detect the expression of matrix metalloproteinase (MMP)-2, MMP-9, and tissue inhibitor of metalloproteinases 1 (TIMP-1) in each cell group. **p < 0.01 compared with control group, ^##^*p* < 0.01 compared with Ang-II group, ^&^*p* < 0.05 compared with Ang-II group, and ^&&^*p* < 0.01 compared with Ang-II group. SENCR: Smooth muscle and endothelial cell enriched migration/differentiation-associated lncRNA.

MMPs and TIMP-1 are the major enzymes related to the degradation of extracellular matrix. An increase of MMP-2 and MMP-9 and a decrease of TIMP-1 indicate extracellular matrix degradation [22,23]. Here, our data showed that overexpression of SENCR significantly inhibited the stimulatory effect of Ang-II on MMP-2 and MMP-9 expression and the inhibitory effect on TIMP-1 expression. However, knockdown of SENCR remarkably facilitated the stimulatory effect of Ang-II on MMP-2 and MMP-9 expression and the inhibitory effect on TIMP-1 expression (Figure 3). Overall, overexpression of SENCR suppressed Ang-II-induced degradation of extracellular matrix of VSMCs, while inhibition of SENCR promoted the degradation of extracellular matrix.

### Overexpression of SENCR inhibited the morbidity and development of AAA

**FIGURE 4:**
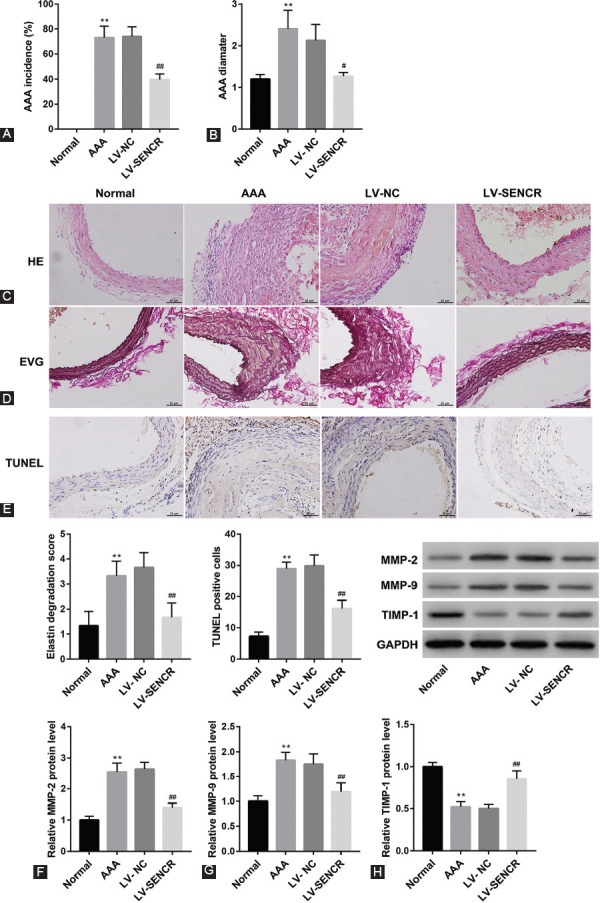
The role of SENCR in abdominal aortic aneurysm (AAA) progression *in vivo*. (A) The incidence rate of AAA was analyzed in 30 mice per each group. (B) The maximum diameter of each aortic tissue was measured. (C) Hematoxylin and eosin staining was performed to detect the pathological change in aortic tissues. (D) Elastica van Gieson staining was carried out to detect the damage of arterial wall elastic fibers. (E) TUNEL staining was performed to measure the cell apoptosis in aortic tissues. (F) Quantification graph showing elastin filament degradation (n = 10 aorta/group). (G) Statistical analysis of TUNEL positive cells. (H) Western blot was performed to detect the expression of matrix metalloproteinase (MMP)-2, MMP-9, and tissue inhibitor of metalloproteinases 1 (TIMP-1) in aortic tissues. **p < 0.01 versus normal group, ^#^*p* < 0.05 versus AAA group, and ^##^*p* < 0.01 versus AAA group. SENCR: Smooth muscle and endothelial cell enriched migration/differentiation-associated lncRNA.

Subsequently, we investigated the role of SENCR in AAA formation. LV carrying pcDNA-SENCR or empty vector were injected into Ang-II-induced ApoE^-/-^ mice (n = 30 per each group). At 28 days later, aortic tissues were isolated from each mice, and then we found that overexpression of SENCR could effectively reduce the incidence of AAA (Figure 4A) as well as reduce the aortic maximum diameter of AAA mice (Figure 4B). Subsequently, 10 aortic tissues were randomly selected from each group for histological analysis. Our results showed that overexpression of SENCR notably attenuated the lesions in the aortic tissues from AAA mouse model (Figure 4C) as well as improved the damage in the arterial wall elastic fibers (Figure 4D and F). In addition, the TUNEL staining results showed that overexpression of SENCR could suppress Ang-II-induced cell apoptosis in aortic tissues (Figure 4E and G). Furthermore, consistent with the *in vitro* results, overexpression of SENCR could effectively inhibit the expression of MMP-2 and MMP-9 and facilitate the expression of TIMP-1 in the aortic tissues from AAA mice (Figure 4H). Taken together, overexpression of SENCR notably inhibited AAA formation and development through inhibiting apoptosis and extracellular matrix degradation of VSMCs.

## DISCUSSION

AAA is an acquired, immune-driven destruction of the aortic wall, and AAA rupture leads to high mortality in older adults. However, due to the lack of effective drugs and a less invasive treatment method, endovascular aortic repair, instead of traditionally open surgical repair, has gradually become a major therapeutic method of AAA [24]. To find an effective medical therapy of AAA is a major challenge that requires an in-depth understanding of the pathophysiology of AAA. Inflammation, SMC apoptosis, extracellular matrix degradation, accumulation of T lymphocytes, and oxidative stress are important pathological features of AAA [25,26]. In this present study, we focused on the effect of SENCR on the apoptosis and extracellular matrix degradation of VSMCs.

The crucial role of lncRNAs in gene expression determines their important regulatory function in various pathological processes, such as malignant tumors, cardiovascular and neurodegenerative diseases [27,28]. SENCR, also named as FLI1-AS1 or lncRNA9, was proved to play an important role in the stabilization of differentiated state and contractile phenotype of SMCs. Some researchers demonstrated that SENCR is highly expressed in endothelial cells, SMCs, and aortic tissues under the physiological conditions [15,29,30]. However, SENCR was proved to be downregulated in the plasma of patients with coronary heart disease. Upregulation of SENCR could effectively inhibit the stimulatory effect of platelet-derived growth factor (PDGF)-BB on human aortic VSMC proliferation and migration [31]. SENCR may be involved in the development of AAA. In this present study, we found that SENCR was downregulated in AAA tissues and Ang-II-induced VSMCs. Importantly, our results showed that overexpression of SENCR inhibited AAA formation, and attenuated the damage in aortic tissue *in vivo* and Ang-II-induced VSMC apoptosis.

MMPs are a family of zinc-dependent endopeptidases, and a key class of proteases in the degradation of extracellular matrix. MMPs could effectively accelerate the development of AAA through degradation of elastic and collagen fibers in aortic tissues, especially MMP-2 and MMP-9, which were considered as key regulators in the formation and development of AAA [32]. Morris et al. indicated that MMPs are upregulated in the aortic tissues from patients with AAA, while the inhibitor of MMPs – TIMP-1 is downregulated in these aortic tissues [33]. TIMP-1 is another important regulator of extracellular matrix degradation. In AAA, apoptosis of VSMCs is closely associated with increased MMPs and decreased TIMP-1 [34,35]. These factors together promote the progression of AAA. In this present study, our data proved that MMP-2 and MMP-9 were increased both in AAA tissues and Ang-II-stimulated VSMCs, while TIMP-1 was decreased in AAA. However, overexpression of SENCR could obviously decrease the expression of MMP-2 and MMP-9 and increase the expression of TIMP-1. Overexpression of SENCR could inhibit extracellular matrix degradation induced by Ang-II. Nevertheless, inhibition of SENCR could aggravate the effect of Ang-II on AAA formation and VSMC apoptosis.

## CONCLUSION

Overall, in this present study, we showed for the first time that overexpression of SENCR could remarkably prevent Ang-II-induced formation of AAA in ApoE^-/-^ mice by inhibiting VSMC apoptosis and degradation of extracellular matrix. Upregulation of the lncRNA SENCR may be a potential strategy for preventing AAA formation and development. However, more work needs to be done. The action mechanism of SENCR in regulating VSMC apoptosis and extracellular matrix degradation in AAA remains unclear.
